# Supplementary material for: Engineering functionality-optimized fully human B7-H3 CAR T cells for enhanced solid tumor therapy
Source: Cell Rep Med. 2026 Apr 29;7(5):102782. doi: 10.1016/j.xcrm.2026.102782 (PMC13198247; doi:10.1016/j.xcrm.2026.102782)
Supplement: Document S1. Figures S1–S7 [file mmc1.pdf]

Cell Reports Medicine, Volume *7*

Supplemental information

Engineering functionality-optimized fully human B7-H3 CAR T cells for enhanced solid tumor therapy

Pradip Bajgain, Yang Feng, Mariela Puebla, Meijie Tian, Kuo-Sheng Hsu, Jaewon Lee, GuoJun Yu, Liping Yang, Steven Seaman, Mary Beth Hilton, Karen Morris, Niza Borchin, Jennifer D. Tran, Riley D. Metcalfe, Dan Li, Mitchell Ho, James C. Cronk, Javed Khan, Anandani Nellan, Rosandra N. Kaplan, and Brad St. Croix

A

scFv clone	Sequences
**Y868**	**V_H Sequence**<br>CDR1 CDR2 CDR3<br>QVQLVESGGGLVQPGGSLRLSCAASGFTFSSYAMSWVRQAPGKGLEWVSAISGSGGSTYYADSVKGRFTISRDNAKNSLYLQVNSLRAEDTALYYCVRDTYRFFDYWGQGTLVTVSS<br>**V_L Sequence**<br>CDR1 CDR2 CDR3<br>QSVLTQPPSVSGAPGQRVTISCSGSTSNIGARYPVHWYQQFPGTVPKLLIYGNNNRPSGVPDRFSGSKSGTSASLVITGLQAGDEADYYCQSFDNSLSGVLFGGGTKVTVLG
**Y111**	**V_H Sequence**<br>CDR1 CDR2 CDR3<br>QVQLVQSGAEVKKPGASVKVSCKASGYTFTSYGISWVRQAPGQGLEWMGRIIPILGIANYAQKFQGRVTITADKSTSTAYMELSSLRSEDTAVYYCARSNPGGAFDIWGQGTMVTVSS<br>**V_L Sequence**<br>CDR1 CDR2 CDR3<br>DIQLTQSPSTLSASVGDRVTITCRASQSVRSNLAWYHQKPGKAPKLLIYKASTLESGVPSRISGSGSGTEFTLTISSLQPDDFATYFCQQYAYYPTFGQGTRLEIKG
**Y117**	**V_H Sequence**<br>CDR1 CDR2 CDR3<br>QVQLVQSGAEVKQPGASVKVSCKASGYTFTDYAITWVRQAPGQGLEWVGWINTDTGNPTYAQAFTGRLVFSLDTSVSTAYLQISSLKAEDTAVYYCARVTPSGSLVHYDFWGQGTLVTVSS<br>**V_L Sequence**<br>CDR1 CDR2 CDR3<br>QSVLTQPPSVSGAPGQRVTISCTGSRSNTGAGFDVHWYQQLPGTAPKLLIYGNNNRPSGVPDRFSGSRSGTSASLAINGLQAEDEADYYCQSYDYNLSGVIFGGGTKLTALG

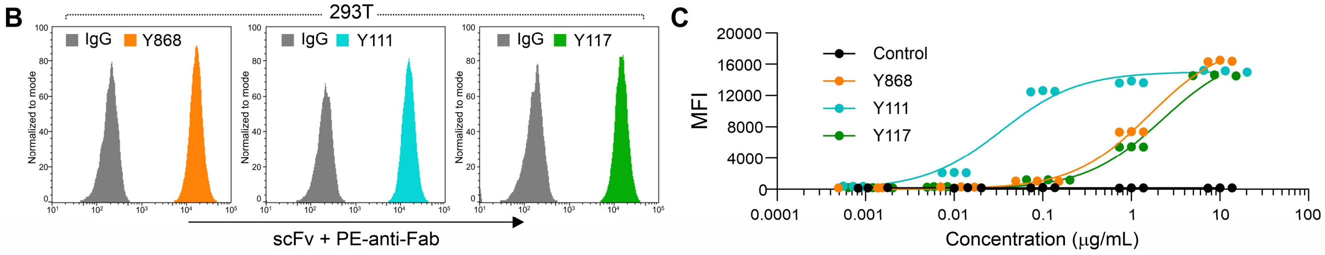

Figure S1. B7-H3 antibodies contain distinct variable domains and bind specifically to B7-H3. Related to Figure 1.

(A) Variable domain sequences of Y868, Y111 and Y117.

(B) Flow cytometry evaluating binding of Y868, Y111 and Y117 to 293T cells.

(C) Flow cytometry showing the dose-dependent binding of each of the scFvs. MFI: mean fluorescence Intensity. Replicates were staggered on the x-axis to visualize individual samples because inter-replicate variability was minimal.

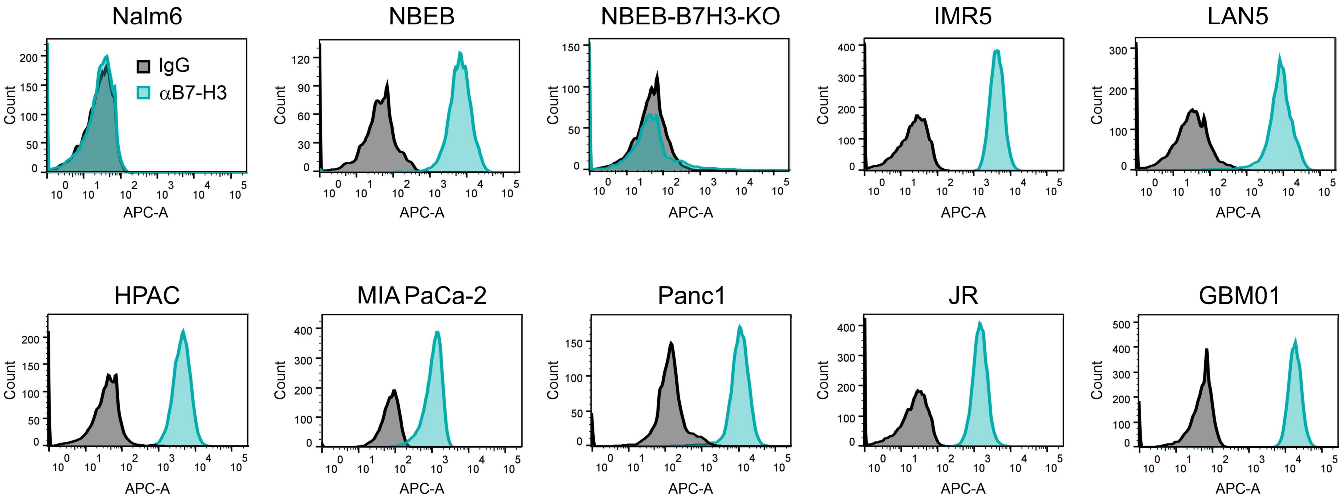

Figure S2. B7-H3 expression on cancer cell lines used in this study. Related to Figure 2.
Flow cytometry was used to evaluate B7-H3 expression on neuroblastoma (NBEB, IMR5 and LAN5), pancreatic cancer (HPAC, MIA PaCa-2, Panc1), rhabdomyosarcoma (JR) and pediatric glioblastoma (GBM01) cell lines. Nalm6 leukemia cells and NBEB-B7H3 KO cells were used as specificity controls. IgG: non-binding IgG isotype control.

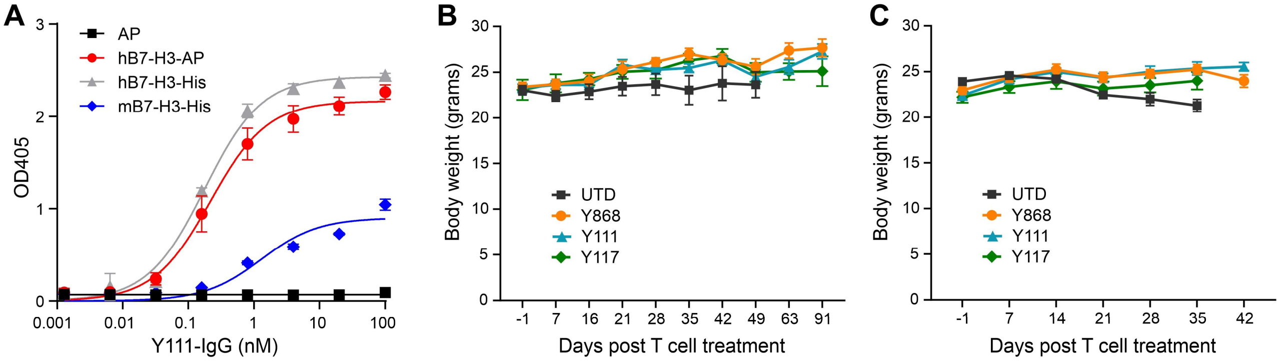

Figure S3. B7-H3 CAR T cells are well tolerated in vivo. Related to Figure 4.
(A) ELISA showing reactivity of Y111 full IgG to Alkaline phosphatase (AP) control protein, or the extracellular domain (ED) of human B7-H3 fused to AP (hB7-H3-AP) or 6xHIS (hB7-H3-His), or mouse B7-H3 ED fused to 6xHIS (mB7-H3-His). Data represent mean ± SD.
(B) Body weight measurements following CAR T treatment for mice in the Panc1 study shown in Figure 4A-D of the main text. Data represent mean ± SEM.
(C) Body weight measurements following CAR T treatment for mice in the HPAC study shown in Figure 4E-G of the main text. Data represent mean ± SEM.

A

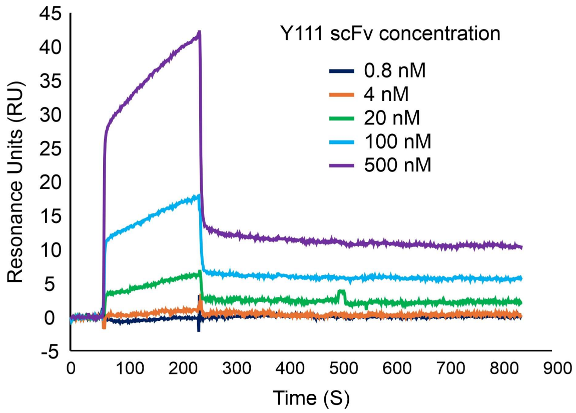

		human B7H3-ED (4Ig)			mouse B7H3-ED (2Ig)		
	mAb	ka (1/Ms)	kd (1/s)	KD (nM)	ka (1/Ms)	kd (1/s)	KD (nM)
IgG	Y111	1.2×10^{6}	2.6×10^{-2}	21.1	1.4×10^{5}	0.05	366.2
	MGA271	1.9×10^{5}	1.1×10^{-3}	5.7	ND	ND	ND
	376.96	5.3×10^{4}	1.1×10^{-3}	20.9	1.0×10^{4}	0.02	2040.1
scFv	Y111	4.3×10^{4}	5.0×10^{-4}	11.8	-	-	-
	MGA271	1.7×10^{5}	1.5×10^{-2}	91.0	-	-	-
	376.96	1.7×10^{5}	2.0×10^{-2}	116.9	-	-	-

ND: no detectable binding

Figure S4. Affinity measurements for Y111, MGA271, and 376.96 antibodies. Related to Figure 5.
(A) Biacore sensogram showing the binding kinetics of Y111 scFv to immobilized human B7-H3-4Ig extracellular domain (ED). Y111 exhibits rapid initial binding, followed by a second, concentration-dependent association phase. B7-H3 contains an internal repeat within its ED, and Y111 preferentially forms a 1:1 complex with B7-H3 (see Fig. S7D). The weaker secondary association likely reflects binding of a second scFv at the unoccupied repeat in the 1:1 complex at high Y111 scFv concentrations. During the dissociation phase, the resonance units (RU) decrease rapidly, followed by a slower decline, consistent with rapid dissociation from the secondary, lower-affinity site and slower release from the primary binding site.
(B) Summary of binding affinities of Y111, MGA271, and 376.96 in either full IgG (bivalent) or scFv (monovalent) formats against B7-H3 ED. Y111 is unusual in that it exhibits higher affinity in monovalent scFv format, indicating that avidity of the antibody does not contribute substantially to its binding strength.

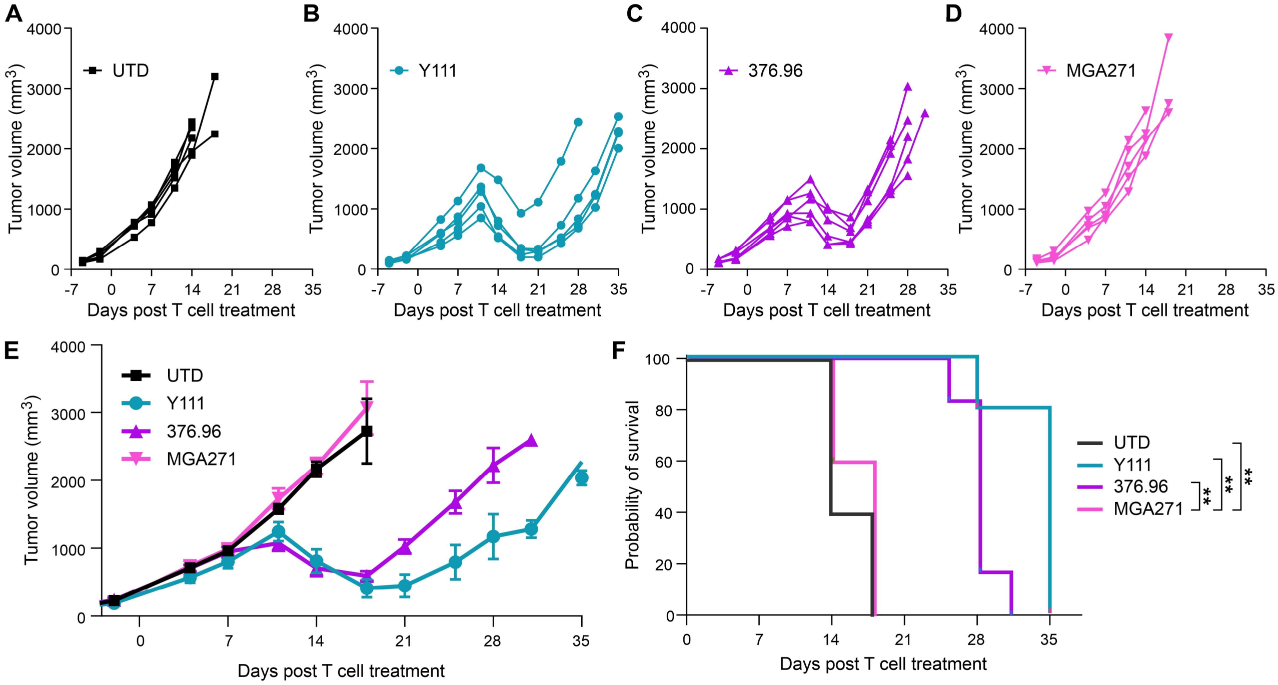

Figure S5. Y111 compares favorably with 376.96-, MGA271- or B12-based CARs in a rhabdomyosarcoma model. Related to Figure 6.

(A-D) Individual tumor growth curves of JR-1 rhabdomyosarcoma following intramuscular tumor implantation and subsequent i.v. administration of (A) UTD control, (B) Y111, (C) 376.96, or (D) MGA271 CAR T cells. n=5-6 per group.

(E) Mean tumor growth curves for the treatment groups shown in (A). Data shown as mean ± SEM.

(F) Kaplan–Meier survival curves corresponding to the study in (A) to (E). Statistical significance was assessed using a log-rank (Mantel–Cox) test. n=5 (UTD, Y111, and MGA271) or 6 (376.96). P values were from a log-rank (Mantel-Cox) test. n=5-6/group. **P<0.01.

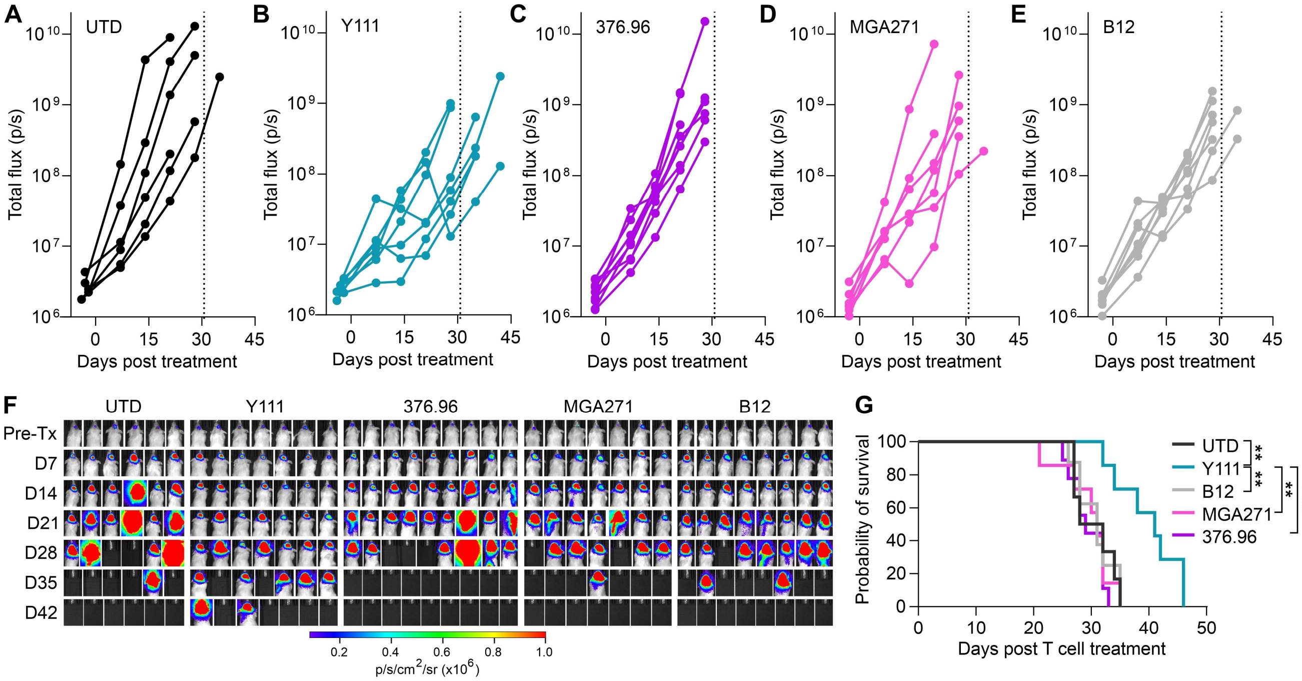

Figure S6. Y111 compares favorably with 376.96-, MGA271- or B12-based CARs in a glioblastoma model. Related to Figure 6.

(A-E) BLI was used to monitor growth of luciferase labeled GBM01 glioblastoma following stereotactic orthotopic implantation of tumor cells and subsequent i.v. administration of (A) UTD control, (B) Y111, (C) 376.96, (D) MGA271 or (E) B12 CAR T cells. Groups were randomized for treatment based on an equivalent tumor luminescence. Each line represents an independent animal. n=6-9 per group.

(F) Images from the study shown in (A) to (E).

(G) Kaplan–Meier survival curves corresponding to the study in (A) to (F). Statistical significance was assessed using a log-rank (Mantel–Cox) test. n=6 (UTD), 7 (Y111 and MGA271), 8 (B12), or 9 (376.96). **P<0.01, ***P<0.001.

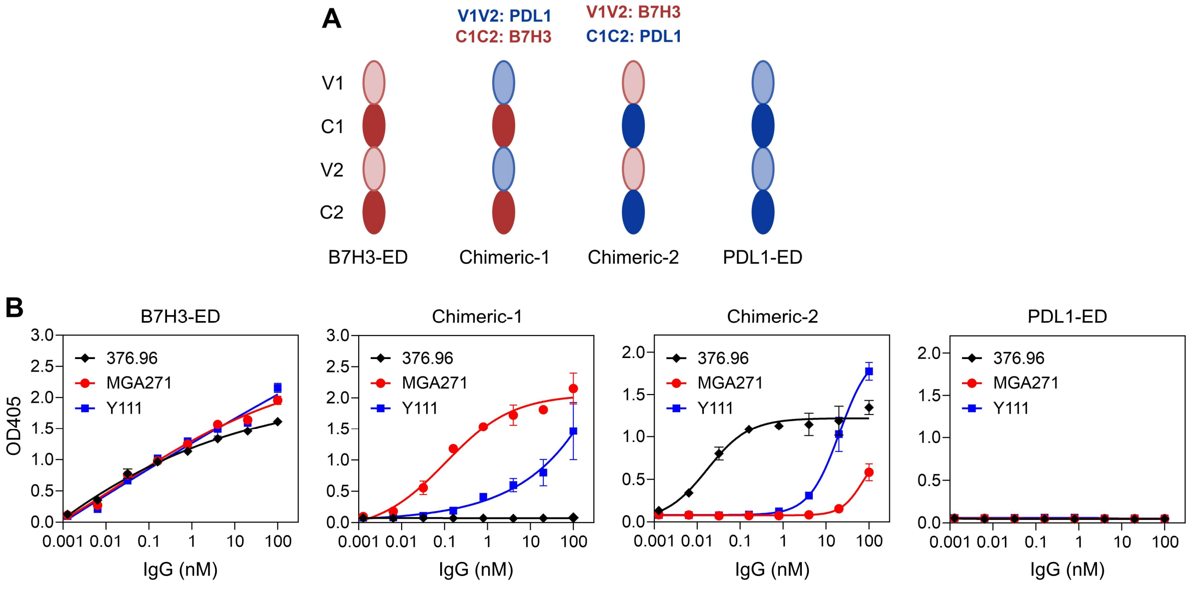

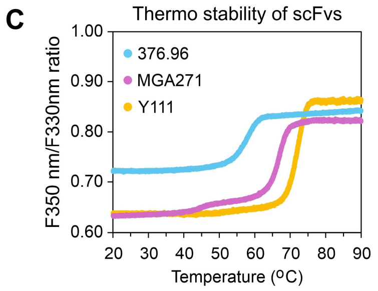

D

Observed composition of B7H3-ED/Ab IgG complex at different input ratios

mAb	B7H3-ED : full IgG input ratio 1:1	1:2	1:4	Ab epitope location
376.96	1:2 (73%)	1:2 (55%)	2:2 (64%)	IgV domain
MGA271	2:2 (100%)	2:2 (100%)	2:2 (100%)	IgC domain
Y111	1:1 (100%)	1:1 (100%)	1:1 (68%), 1:2 (12%)	IgC and IgV domain

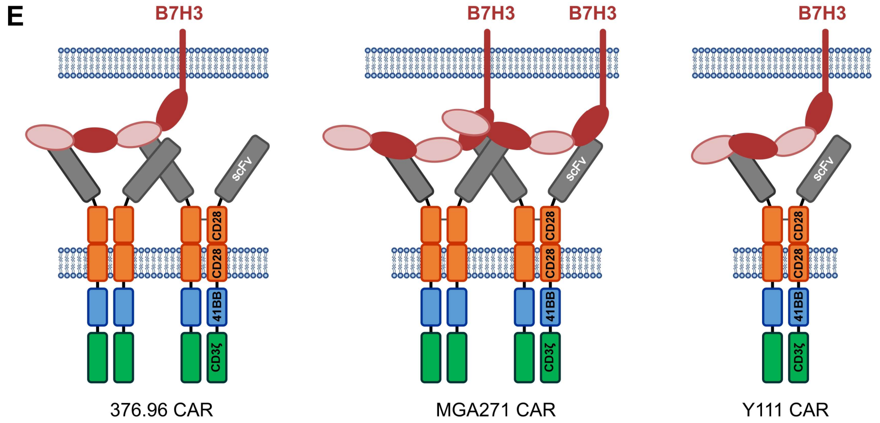

Figure S7. Y111 displays unique binding kinetics.
(A) Schematic overview of B7-H3 and PDL1 extracellular domain (ED) fusion proteins used in domain-swap experiments to map the binding domain of the 376.96, MGA271 and Y111 antibodies.
(B) ELISA was used to evaluate binding of the 376.96, MGA271 and Y111 full IgG antibodies to the purified fusion proteins depicted in (A). Data shown as mean ± SD.
(C) Thermal stability of the 376.96, MGA271 and Y111 scFvs.
(D) SEC-MALS was used to determine B7-H3-ED/full IgG complex formation following mixture at different input ratios. Note that the bivalent Y111 antibody strongly prefers 1:1 binding even in the presence of an excess of Y111, consistent with the Biacore data of Figure S4A.
(E) Schematic overview showing how the bivalent Y111 CAR may preferentially bind the 4Ig form of B7H3 - soluble or membrane bound as shown - in a 1:1 complex.
